# Impact of tumor attachment to the pleura measured by a pretreatment CT image on outcome of stage I NSCLC treated with stereotactic body radiotherapy

**DOI:** 10.1186/s13014-015-0343-6

**Published:** 2015-02-07

**Authors:** Takaya Yamamoto, Noriyuki Kadoya, Yuko Shirata, Masashi Koto, Kiyokazu Sato, Haruo Matsushita, Toshiyuki Sugawara, Rei Umezawa, Masaki Kubozono, Yojiro Ishikawa, Maiko Kozumi, Noriyoshi Takahashi, Kengo Ito, Yu Katagiri, Ken Takeda, Keiichi Jingu

**Affiliations:** Department of Radiation Oncology, Tohoku University School of Medicine, Sendai, Japan; Research Center for Charged Particle Therapy, National Institute of Radiological Sciences, Chiba, Japan; Radiation Technology, Tohoku University Hospital, Sendai, Japan

**Keywords:** Stereotactic radiotherapy, Lung cancer, Prognostic factor, Pleural invasion, Pleural attachment, Pleural contact

## Abstract

**Background:**

Pleural invasion status is known to be a predictor of survival after pulmonary resection for non-small cell lung cancer. Our goal was to determine whether the length of tumor attachment to the pleura on a pretreatment CT image has prognostic value as an alternative to pleural invasion status for stage I non-small cell lung cancer treated with stereotactic body radiotherapy (SBRT).

**Methods:**

A total of 90 tumors in 87 patients (males: 68, females: 19) who received SBRT between March 2005 and September 2011 in our institution were reviewed. The median age of the patients was 78 years (range, 48-90 years). The median tumor diameter was 2.2 cm (range, 0.9-4.2 cm). The prescribed dose was typically 48 Gy in 4 fractions, 60 Gy in 8 fractions or 60 Gy in 15 fractions to the isocenter with 6 MV X-ray using 4 non-coplanar and 3 coplanar static beams. The lengths of attachment were measured using pretreatment CT images at the lung window. Cumulative incidence rates were calculated using Kaplan-Meier curves, and univariate and multivariate analyses for in-field tumor control, locoregional control (LRC), freedom from distant metastasis and freedom from progression (FFP) were performed using a Cox proportional hazards model.

**Results:**

Of the 90 tumors, 42 tumors were attached to the pleura (median, 14.7 mm; range, 4.3-36.0 mm), 21 tumors had pleural indentation and 27 tumors had no attachment. The median follow-up period for survivors was 46.1 months. The 3-year in-field control, LRC, FFP and overall survival rates were 91.2%, 75.3%, 63.8% and 68.6%, respectively. SBRT dose and tumor diameter were independently significant predictors of in-field control (p = 0.02 and p = 0.04, respectively). Broad attachment to the pleura, the length being more than 14.7 mm, was a negative independent predictor of LRC and FFP (p = 0.02 and p = 0.01, respectively).

**Conclusions:**

Pleural attachment status on a pretreatment CT image might be an important predictor of LRC and FFP.

## Background

Among the various lung cancer treatment options, surgical resection has been the standard treatment for early-stage non-small cell lung cancer (NSCLC), and relationships between treatment outcomes and clinical, operative or pathological findings have been reported. Based on these findings, the staging system has been revised and the Union Internationale Contre le Cancer (UICC) TNM classification of malignant tumors 7th edition is now in worldwide use [1,2]. According to the TNM classification for lung cancer, pathological findings of visceral pleural invasion beyond the elastic layer (pl1), invasion to the pleural surface (pl2) or invasion to the interlobar pleura (pl3) is upgraded to T2a and therefore upstaged to stage IB even if the tumor diameter is 3 cm or less. In contrast, a tumor of 3 cm or less in diameter with superficial pleural invasion beneath the elastic layer (pl0) remains T1 and therefore remains stage IA. This precise definition of visceral pleural invasion referring to the status of the elastic layer is one of the changes from the TNM 6th edition to the TNM 7th edition [3]. Reports about the prognostic value of visceral pleural invasion after a lung operation have been published since the revision, and there has been a report about the relationship between presurgical computed tomography (CT) images and pathological difference: pl1, pl2 and pl3 [4-6].

On the other hand, stereotactic body radiotherapy (SBRT) is a relatively new treatment. However, with progress in the SBRT technique, better treatment outcomes and higher local control rate for stage I NSCLC have been reported [7-11]. Although a lung operation is a standard treatment for early-stage NSCLC, SBRT is often chosen alternatively as a curative treatment if operative risks are expected to overcome the benefit [12]. SBRT is an important treatment option for medically inoperable patients and for patients who have refused surgery [13]. For further progress in the technique, many prognostic factors from clinical and technological points of view have been reported. For instance, T stage, standardized uptake value (SUVmax) on 18 F-fluorodeoxyglucose positron emission tomography and biological effective dose (BED) have been reported as predictors of outcomes [9-11,14-16].

The prognostic significance of pleural invasion status after treatment of stage I NSCLC with SBRT has rarely been discussed because gross pathology or microscopic pathology cannot be clarified as in an operation. By taking surgical outcomes into consideration, tumor invasion to the pleura beyond the elastic layer probably affects the treatment outcome and might be a predictor of outcome after SBRT, although there has been only one report about pleural contact to the pleura on a CT image [17]. Although the pathological pleural invasion status cannot be determined in SBRT, we hypothesized that the length of tumor attachment to the pleura on a pretreatment CT image may be an alternative to pathological findings, with broad attachment to the pleura representing tumor invasion beyond the elastic layer, and may have prognostic value. The purpose of this study was to investigate the impact of tumor attachment to the pleura on outcome of stage I NSCLC treated with SBRT by measuring the attachment length with a pretreatment CT image.

## Methods

### Patients

We reviewed our clinical retrospective database, and 110 patients (114 lesions) with stage I NSCLC were treated with SBRT form March 2005 through November 2011. Patients with follow-up of less than 3 months, patients with tumors having mainly a ground glass opacity component and patients with tumors that could not be measured on axial and coronal sections of pretreatment CT images were excluded. Ninety tumors in 87 consecutive patients were used for analysis in this study. The main clinical and pathological characteristics are summarized in Table 1. This retrospective study was approved by the Ethical Committee of Tohoku University Hospital (No. 2011-228) and informed consent was obtained from all patients.

**Table 1:** **Patients and tumor characteristics**

**Characteristic**	**No. (%)**
Age, median, years	78 (range: 48-92)
Gender	
Female	19 (21)
Male	68 (78)
ECOG performance status	
0-1	71 (81)
2	12 (13)
3	4 (4)
Tumor diameter, median, cm	2.2 (range: 0.9-4.2)
≤ 2.0	38 (42)
2.1-3.0	40 (44)
3.1-5.0	12 (13)
Pathology	
Adenocarcinoma	32 (35)
Squamous cell carcinoma	20 (22)
Others	8 (8)
Clinically diagnosed	30 (33)
SBRT dose, BED_10_	
≤ 102 Gy_10_	44 (48)
> 102 Gy_10_	46 (51)
Prescription	
Isocenter	59 (65)
D95	31 (34)

Data are shown as number (percentage) of patients for parameters from age to performance status and as number (percentage) of tumors for other parameters.

Abbreviations: ECOG, Eastern cooperative oncology group; BED_10_, biological effective dose, calculated by the formula BED = nd [1 + d/(α/β)], where n is the number of fractions, d is the dose/fraction, and α/β ratio is 10 Gy for tumors; D95, doses covering 95% of the planning target volume.

### Pretreatment CT images and measurements

Chest imaging for all tumors was performed with a multidetector CT scanner. In our institute, thoracic CT scanning has usually been performed using SOMATOM Definition (Siemens, Forchheim, Germany), Aquilion 64 (Toshiba Medical Systems, Nasushiobara, Japan) or Robusto (Hitachi Medico, Tokyo, Japan) with breath hold and with or without injection of an intravenous contrast material because the attachment lengths of all tumors were measured at window settings for the lung (center, -700 HU; width, 1500 HU). The scanning parameters were 120 kVp and an auto milliampere setting (100-220 milliamperes). Slice thickness ranged from 1.0 mm to 10 mm, and the median and mean slice thicknesses were 2.0 mm and 2.5 mm, respectively.

**Figure 1:**
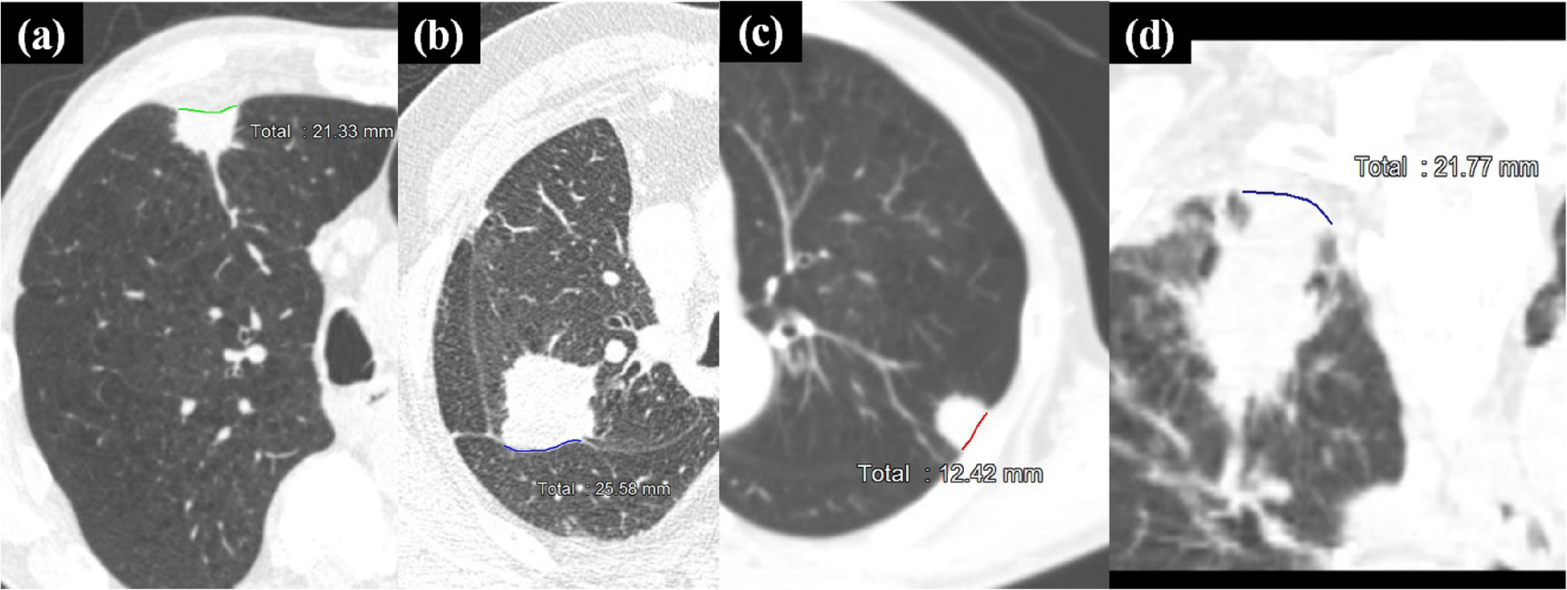
**Length of tumor attachment to the pleura were measured at the lung window.** Measurements of tumor attachment to the visceral pleura **(a)**, tumor attachment to the interlobar pleura **(b)**, tumor attachment to the pleura in non-high-resolution CT **(c)**, tumor attachment to the pleura at a coronal section **(d)**.

Positive pleural attachment was defined as tumor attachment to the visceral pleura on CT images at both the lung window and mediastinal window (center, 45 HU; width, 320 HU) or tumor attachment to the interlobar pleura at the lung window. Positive pleural indentation was defined as tumor indentation to the visceral pleura on CT images at the lung window. Maximum tumor diameter, tumor attachment and indentation to the pleura, and length of tumor attachment to the pleura were measured using AquariusNET Viewer (TeraRecon, Foster City, CA) at the lung window with axial and coronal sections. The length of tumor attachment was the sum of measurements of short lines along the pleural attachment surface (Figure 1).

### SBRT procedure

We previously reported details of the SBRT technique [18]. The patient was immobilized in the supine position in a body frame (Vac-loc, Med-tek, Orange City, IA) with or without an abdominal pressure belt based on tumor location and tumor (or implanted gold marker) breathing motion on fluoroscopic movies by a simulator (Ximatron, Varian Medical Systems, Palo Alto, CA), and tumor (or implanted gold marker) breathing motion was subsequently measured. Then a slow-rotation serial CT scan in the same position was performed at intervals of 2.5 mm and acquisition time of 4 seconds. Internal margins were formed by both the motion measurement on fluoroscopy and tumor blurring on a slow-rotation CT. Gross tumor volume (GTV) was defined as the visible extent of the tumor on a slow-rotation scanning CT image at the lung window, and clinical target volume was defined as GTV plus a 0-5 mm margin for microscopic invasion. For set-up uncertainty, set-up margins of 5 mm and daily on-board imager (Varian Medical Systems, Palo Alto, CA) were required. Treatment was performed using 6 MV photons with 5 to 7 coplanar or non-coplanar multi-static beams. The SBRT plan was created with the Eclipse planning system (Varian Medical Systems, Palo Alto, CA).

Before June 2009, the pencil beam method with heterogeneity correction (modified Batho power law) was utilized for planning, and 48 Gy in 4 fractions, 60 Gy in 8 fractions or 60 Gy in 15 fractions was delivered to the isocenter. After June 2009, an analytical anisotropic algorithm (AAA) was utilized for planning, and 40 Gy in 4 fractions or 50 Gy in 8 fractions covering 95% of the PTV (D95) was delivered. The prescription dose of 40 Gy in 4 fractions or 50 Gy in 8 fractions is similar to 48 Gy in 4 fractions or 60 Gy in 8 fractions to the isocenter, respectively. The choice of dose depended on tumor location or performance status. Eight or 15 fractions were selected if the lung tumor was adjacent to critical organs such as the main bronchus, heart and esophagus but not the chest wall. SBRT was performed on consecutive treatment days. The isocenter doses were recalculated with AAA (version 11031), and then BED_10_ was calculated using the following formula: BED_10_ = nd [1 + d/(α/β)], where n is the number of fractions, d is the isocenter dose per fraction, and α/β ratio is 10 Gy for the tumor. Median BED_10_ was 102.1 Gy_10_ (range: 74.2-121.0 Gy_10_).

### Follow-up and evaluation

Follow-up procedures have been reported elsewhere [18]. Follow-up examinations by a radiation oncologist were performed every 3-6 months for 2 years after SBRT and then every 6 months. Patients also underwent examinations by physicians. In-field control was defined as tumor control within the 95% isodose line, and locoregional control (LRC) was defined as disease control within the treated lobe and hilar or mediastinal lymph node. The final diagnosis of any control failure was made by physicians and radiation oncologists. Toxicity was graded using the National Cancer Institute Common Terminology Criteria for Adverse Events ver. 4.0.

### Statistical analysis

Time to an event was calculated from the first day of SBRT to the day an event was confirmed. Cumulative in-field control, LRC, freedom from distant metastasis (FFDM), freedom from progression (FFP) and overall survival (OS) were calculated using Kaplan-Meier curves, and two or more Kaplan-Meier curves were compared using log-rank tests. Differences between three or more groups were evaluated by the Kruskal-Wallis test. Univariate and multivariate Cox regression analyses were used to determine whether variables had prognostic value or not. Correlation coefficients for all variables were calculated to avoid multicollinearity. A p-value < 0.05 was defined as significant in all tests. JMP v.11.0.0 (SAS Institute, Cary, NA) was used for statistical analyses.

## Results

### Measurement results

**Figure 2:**
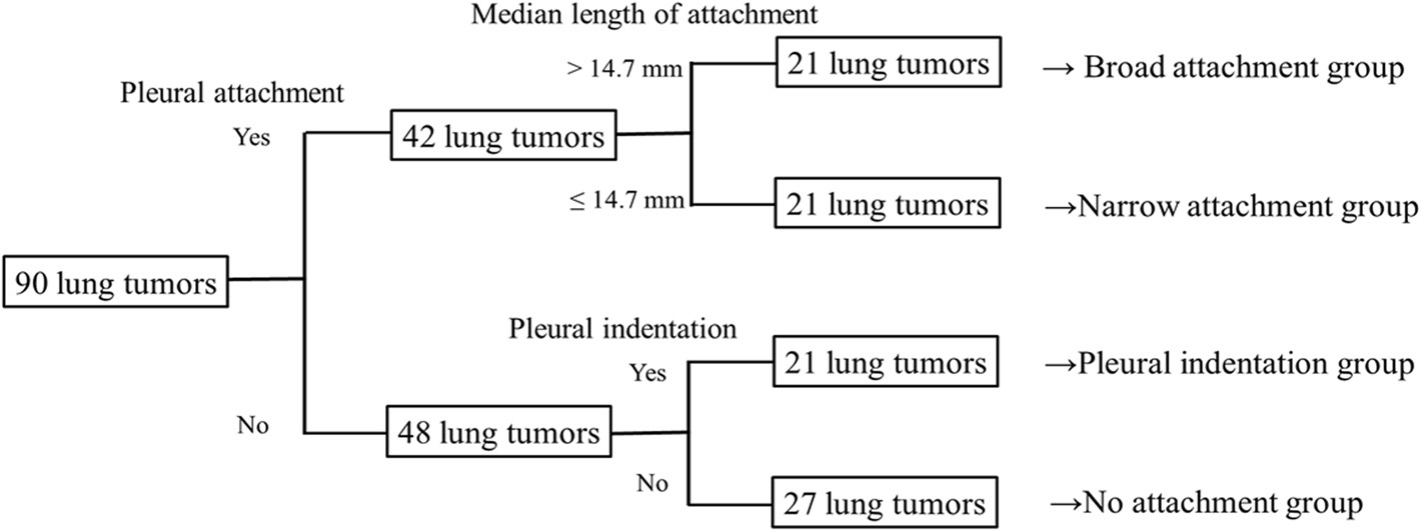
**Ninety lung tumors were divided into four groups according to attachment status.** The median length of positive attachment of 42 tumors was 14.7 mm (range, 4.3-36.0 mm), and the tumors were divided at the sample median into broad attachment and narrow attachment groups.

Among the 90 tumors, 42 tumors were attached to the pleura, 21 had pleural indentation and 27 had neither pleural attachment nor pleural indentation (no attachment group). The median length of attachment of the 42 tumors was 14.7 mm (range, 4.3-36.0 mm), and the tumors were separated into two groups at the median value: a broad attachment group and a narrow attachment group (Figure 2).

### Treatment results

The median follow-up period for survivors was 46.1 months (range: 18.5-90.2 months) and that for all patients was 39.0 months (range: 3.3-90.2 months). During follow-up, there were in-field failure in 7 of the 90 tumors (7.7%), involved lobar failure in 14 of the 89 lobes (15.7%), regional lymph node failure in 12 of the 87 patients (13.7%), locoregional failure in 22 of the 87 patients (25.2%), distant metastasis in 23 of the 87 patients (26.4%) and any failure in 38 of the 87 patients (43.6%). The median time to in-field failure was 26.1 months (range: 8.9-66.9 months), the median time to locoregional failure was 26.7 months (range: 8.7-66.9 months) and the median time to any failure was 28.8 months (range: 3.3-79.5 months). Of 7 in-field failure cases, 5 cases occurred in the broad attachment group, 1 case occurred in the narrow attachment group and 1 case occurred in no attachment group. Three of the 5 failure cases in the broad attachment group and the failure case in the narrow attachment group recurred with attachment to the pleura, and the other three in-field recurrences were in intrapulmonary region. Forty-three patients died during follow-up: 22 died of primary disease and 21 died of other causes. The 3-year in-field control, LRC, FFDM, FFD and OS rates were 91.2% (95% confidence interval [CI]: 81.4-96.0%), 75.3% (95% CI: 63.5-84.2%), 76.3% (95% CI: 64.8-84.9%), 63.8% (95% CI: 51.9-74.3%) and 68.6% (95% CI: 57.8-77.7%), respectively. The median periods of FFDM, FFD and OS were 79.5, 49.1 and 65.0 months, respectively. Pneumonitis of grade 2 occurred in 11 patients, and pneumonitis of grade 3 occurred in 2 patients.

**Figure 3:**
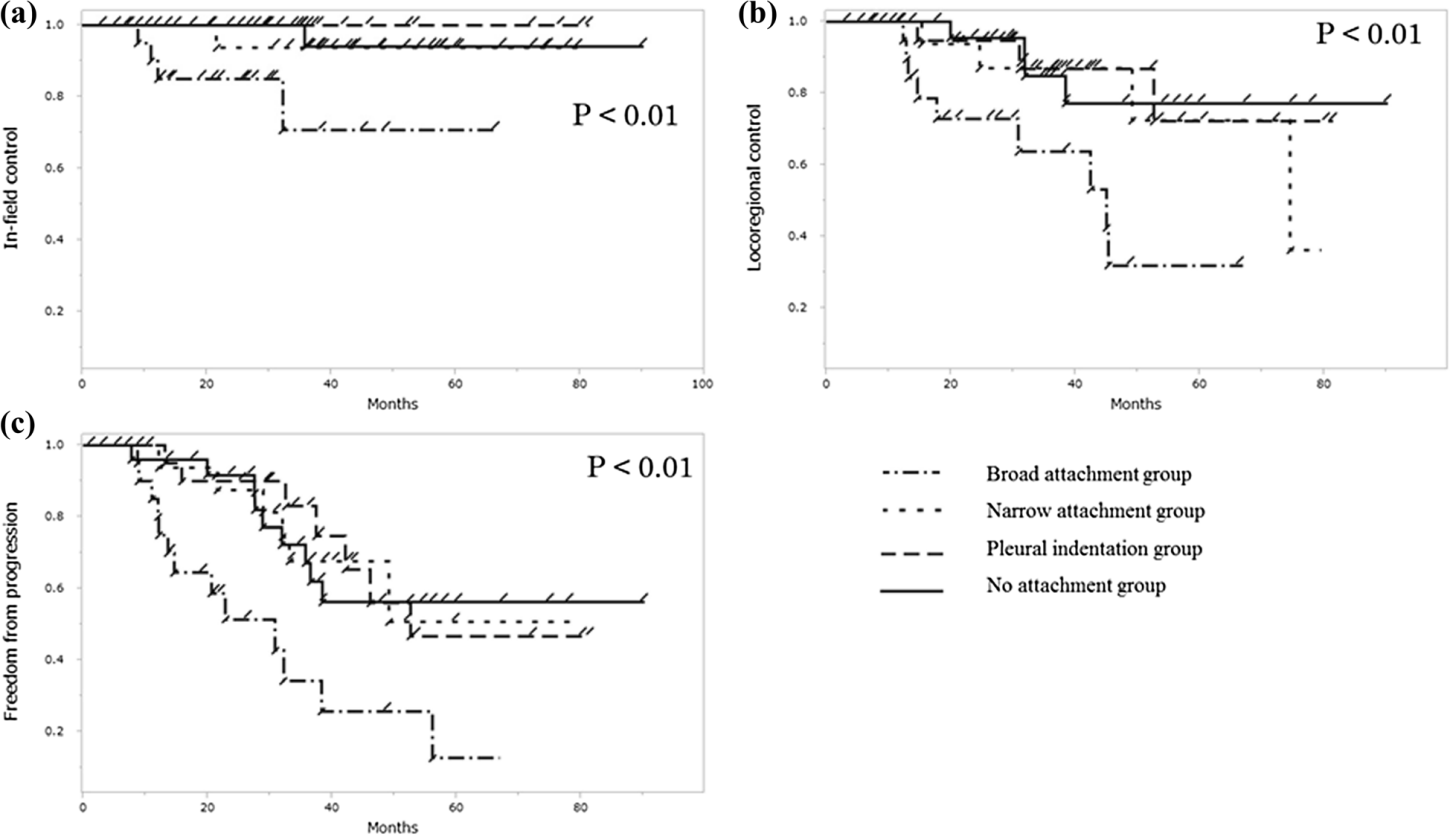
**The broad attachment group showed significantly lower curves in in-field control (a), LRC (b) and FFP (c) than the others (p < 0.01, p < 0.01 and p < 0.01, respectively, log-rank test).**

The median BED_10_ values in the broad attachment, narrow attachment, pleural indentation and no attachment groups were 98.5 Gy_10_ (range: 83.3-107.5 Gy_10_), 102.4 Gy_10_ (range: 82.6-106.9 Gy_10_), 103.3 Gy_10_ (range: 74.2-121.0 Gy_10_) and 101.8 Gy_10_ (range: 91.5-106.0 Gy_10_), respectively (p = 0.06). The broad attachment group had significantly lower in-field control, LRC and FFP rates than those in the narrow attachment group, pleural indentation group and no attachment group (p < 0.01, p < 0.01 and p < 0.01, respectively), but there were no significant differences between the narrow attachment group, pleural indentation group and no attachment group in in-field control, LRC and FFP (p = 0.93, p = 0.45 and p = 0.90, respectively; Figure 3).

### Univariate and multivariate analyses

The results of univariate and multivariate analyses for in-field control, LRC, FFDM and FFD are shown in Table 2. In univariate analyses, attachment status (broad attachment group vs. the others; p < 0.01, hazard ratio [HR]: 11.5, 95% CI: 2.72-81.5), tumor diameter (per 1 cm increase; p < 0.01, HR: 3.71, 95% CI: 1.47-9.72) and SBRT dose (BED_10_: > 102 Gy_10_ vs. ≤ 102 Gy_10_; p = 0.02, HR: 0.13, 95% CI: 0.01-0.79) for in-field control, attachment status (broad attachment group vs. the others; p < 0.01, HR: 4.60, 95% CI: 1.91-10.8) and tumor diameter (per 1 cm increase; p < 0.01, HR: 2.25, 95% CI: 1.30-3.83) for LRC, attachment status (broad attachment group vs. the others; p = 0.01, HR: 3.02, 95% CI: 1.22-7.19) and tumor diameter (per 1 cm increase; p < 0.01, HR: 2.01, 95% CI: 1.19-3.33) for FFDM, and attachment status (broad attachment group vs. the others; p < 0.01, HR: 3.78, 95% CI: 1.87-7.40) and tumor diameter (per 1 cm increase; p < 0.01, HR: 2.02, 95% CI: 1.32-4.81) for FFP were significant predictors. In multivariate analyses, tumor diameter (per 1 cm increase; p = 0.04, HR: 3.65, 95% CI: 1.00-14.1) and SBRT dose (BED_10_: > 102 Gy_10_ vs. ≤ 102 Gy_10_; p = 0.02, HR: 0.10, 95% CI: 0.01-0.75) were significant independent predictors of in-field control, broad attachment was a negative, independent predictor of LRC (p = 0.02, HR: 3.08, 95% CI: 1.15-8.27) and FFP (p = 0.01, HR: 2.66, 95% CI: 1.21-5.73), and no significant factor for FFDM emerged (Table 2).

**Table 2:** **Univariate analysis (upper column) and multivariate analysis (lower column) using Cox regression**

**Variables**	**In-field control**			**Locoregional control**			**Freedom from distant metastasis**			**Freedom from progression**		
	**P value**	**HR**	**95% CI**	**P value**	**HR**	**95% CI**	**P value**	**HR**	**95% CI**	**P value**	**HR**	**95% CI**
Attachment status: broad attachment vs. others	<0.01*	11.5	2.42-81.5	<0.01*	4.60	1.91-10.8	0.01*	3.02	1.22-7.19	<0.01*	3.78	1.87-7.40
Gender: female vs. male	0.89	1.11	0.15-5.20	0.29	0.57	0.16-1.54	0.57	1.29	0.49-3.03	0.54	0.79	0.35-1.62
Diagnosis: pathological vs. clinical	0.17	3.59	0.60-68.1	0.15	2.00	0.78-6.13	0.19	1.81	0.75-5.09	0.11	1.79	0.87-4.04
Tumor diameter (cm): per 1 cm increase	<0.01*	3.71	1.47-9.72	<0.01*	2.25	1.30-3.83	<0.01*	2.01	1.19-3.33	<0.01*	2.02	1.32-3.07
Perfomance status: 0-1 vs. 2-3	0.97	0.96	0.15-18.5	0.88	1.09	0.36-4.71	0.38	1.81	0.52-11.3	0.48	1.42	0.56-4.81
SBRT dose (BED_10_): > 102 Gy_10_ vs. ≤ 102 Gy_10_	0.02*	0.13	0.01-0.79	0.59	1.25	0.54-3.05	0.88	0.94	0.45-2.02	0.77	0.90	0.47-1.75
Prescription: isocenter vs. D95	0.53	1.88	0.30-36.3	0.94	0.96	0.35-3.01	0.79	0.88	0.35-2.48	0.27	0.64	0.30-1.45
Attachment status: broad attachment vs. others	0.15	4.00	0.59-35.6	0.02*	3.08	1.15-8.27	0.19	1.93	0.70-5.29	0.01*	2.66	1.21-5.73
Tumor diameter (cm): per 1 cm increase	0.04*	3.65	1.00-14.1	0.12	1.67	0.86-3.10	0.09	1.68	0.91-3.00	0.06	1.59	0.96-2.55
SBRT dose (BED_10_): > 102 Gy_10_ vs. ≤ 102 Gy_10_	0.02*	0.10	0.01-0.75	n.s.			n.s.			n.s		

Abbreviations: D95, doses covering 95% of the planning target volume; BED, biological effective dose; HR, hazard ratio; CI, confidence interval; n.s, not significant. *p < 0.05.

## Discussion

This study was an important and, to the best of our knowledge, the first analysis of the outcomes of SBRT using the length of tumor attachment specificity. Although pleural invasion status has been widely known as a significant predictor for OS after a lung operation, there have been few attempts to analyze the prognostic value of pleural invasion status or an alternative status after SBRT, presumably because gross pathology or microscopic pathology is not possible after SBRT [3-5,17]. Therefore, an alternative method using CT images in which the length of tumor attachment to the pleura was measured at the lung window was used in this study.

Tumors with broad attachment to the pleura showed significantly worse in-field control, LRC, FFDM and FFD in univariate analysis and the log-rank test (Table 2, Figure 3). In multivariate analysis for in-field control, LRC, FFDM and FFD, a tumor with broad attachment was a negative independent predictor of LRC and FFD. These results indicated that length of tumor attachment to the pleura measured with pretreatment CT images at the lung window might be an important prognostic factor. However, no significant independent factor for FFDM was found, and since 21 of the 87 patients died of other causes, the follow-up periods were thus shortened. More patients and longer follow-up are needed for further analyses.

Although Kaplan-Meier in-field control curves were significantly lower in the broad attachment group, only SBRT dose and tumor diameter were independent prognostic factors for in-field tumor control. This would be because patients in the broad attachment group tended to be prescribed lower SBRT doses at the isocenter. The results for prognostic significance of SBRT dose and tumor diameter confirmed previous findings [8,15]. In-field tumor control was influenced by SBRT dose and tumor diameter, and therefore dose escalation for larger tumors was resonable. In addition, although no significant factor for involved lobar failure emerged, the broad attachment group tended to show lower involved lobar control in univariate analysis (p = 0.06, HR: 3.08, 95% CI: 0.92-9.36). This result possibly indicates that tumors with broad attachment should be considered larger target volumes.

It is important to distinguish stage IA from IB because adjuvant chemotherapy is sometimes considered after surgical resection for stage IB NSCLC [19]. Our results showed that broad attachment was an independent unfavorable predictor for LRC and FFP, and this suggested that tumors with broad attachment might also be regarded as stage IB and considered for additional chemotherapy after or concurrent with SBRT. Although most of our patients were elderly (median age, 78 years), additional chemotherapy would be beneficial even for elderly patients because concurrent chemoradiotherapy for stage III elderly patients (median age, 77 years) using daily low-dose carboplatin showed a survival benefit compared with radiotherapy alone [20]. Therefore, concurrent or adjuvant chemotherapy would be beneficial for improvement of LRC and FFP.

It has been shown that attachment length-to-maximum tumor diameter ratios on presurgical CT images favorably reflected pathological difference: pl1, pl2 and pl3 [6]. However, our study showed that attachment length to the pleura was more discriminative for LRC than attachment length-to-maximum tumor diameter ratio (p = 0.0001 and p = 0.0090, respectively, log-rank test). This difference would arise from the fact that our study aimed to distinguish pl1 from pl0 in stage I NSCLC. Thus, although our results were not based on pathological findings, measurement of tumor attachment to the pleura would be helpful for treatment of SBRT.

No significant differences for in-field tumor control, LRC and FFP were seen between the no attachment group, narrow attachment group and pleural indentation group (Figure 2). In surgical studies, lung cancer with pleural indentation was sometimes excluded from a limited pulmonary resection because of its invasiveness to the pleura [21,22]. However, our study did not show the importance of pleural indentation. Since SBRT was shown to be as effective as sublobectomy or possibly lobectomy using propensity-score matched analysis, pleural indentation status might not be an issue in SBRT [23,24]. The spread of a low dose outside the target volume may be effective. Our inclusion criteria for eliminating mainly ground glass component tumors, short follow-up periods or some non-high-resolution CT images might have hindered the prognostic value of pleural indentation. A tumor with narrow attachment to the pleura was not a significant factor unlike a tumor with broad attachment. Narrow attachment would reflect tumor invasion to the pleura beneath the elastic layer, or would seem attaching to the pleura by partial volume effect of CT images.

This study had some limitations. First, this study was a retrospective, single institute analysis with a limited sample size. Second, high-resolution CT images were available for only 37 of the 90 tumors and therefore the cut-off point in this study was limitative. Ideally, to find a reliable cut-off point, only high-resolution CT images with a sufficient sample size should be used and a receiver operating characteristic curve should be plotted. Finally, various treatment protocols were included in the analysis.

## Conclusions

In conclusion, pleural attachment status on a pretreatment CT image was an important predictor, and broad attachment to the pleura was a negative independent predictor of LRC and FFP. Pleural attachment status might be a good target of additional therapy, but further evidence is needed.

## Abbreviations

NSCLC: Non-small cell lung cancer
UICC: Union internatonale contre le cancer
CT: Computed tomography
SBRT: Stereotactic body radiotherapy
SUVmax: Standardized uptake value
BED: Biological effective dose
ITV: Internal target volume
PTV: Planning target volume
AAA: Analytical anisotropic algorithm
D95: Covering 95% of the PTV
LRC: Locoregional control
FFDM: Freedom from distant metastasis
FFP: Freedom from progression
OS: Overall survival
CI: Confidence interval
HR: Hazard ratio
